# Copy number variations of the ATP-binding cassette transporter *ABCC6* gene and its pseudogenes

**DOI:** 10.1186/1756-0500-5-425

**Published:** 2012-08-09

**Authors:** Marianne K Kringen, Camilla Stormo, Runa M Grimholt, Jens P Berg, Armin P Piehler

**Affiliations:** 1 Department of Pharmacology, Oslo University Hospital, Ullevål, P.O. Box 4956, Nydalen, 0424 Oslo, Norway; 2 Department of Medical Biochemistry, Oslo University Hospital, Ullevål, P.O. Box 4956, Nydalen, 0424 Oslo, Norway; 3 Furst Medical Laboratory, Søren Bullsvei 25, 1051 Oslo, Norway

**Keywords:** Copy number variation, *ABCC6*, Pseudogenes, Pyrosequencing, Transcription

## Abstract

**Background:**

The ATP-binding cassette transporter *ABCC6* gene is located on chromosome 16 between its two pseudogenes (*ABCC6P1* and *ABCC6P2*). Previously, we have shown that *ABCC6P1* is transcribed and affects *ABCC6* at the transcriptional level. In this study we aimed to determine copy number variations of *ABCC6, ABCC6P1* and *ABCC6P2* in different populations. Moreover, we sought to study the transcription pattern of *ABCC6* and *ABCC6* pseudogenes in 39 different human tissues.

**Findings:**

Genomic DNA from healthy individuals from five populations, Chinese (n = 24), Middle East (n = 20), Mexicans (n = 24), Caucasians (n = 50) and Africans (n = 24), were examined for copy number variations of *ABCC6* and its pseudogenes by pyrosequencing and quantitative PCR. Copy number variation of *ABCC6* was very rare (2/142; 1.4%). However, one or three copies of *ABCC6P1* were relatively common (3% and 8%, respectively). Only one person had a single copy of *ABCC6P2* while none had three copies. In Chinese, deletions or duplications of *ABCC6P1* were more frequent than in any other population (9/24; 37.5%). The transcription pattern of *ABCC6P2* was highly similar to *ABCC6* and *ABCC6P1*, with highest transcription in liver and kidney. Interestingly, the total transcription level of pseudogenes, *ABCC6P1* + *ABCC6P2*, was higher than *ABCC6* in most tissues, including liver and kidney.

**Conclusions:**

Copy number variations of the *ABCC6* pseudogenes are quite common, especially in populations of Chinese ancestry. The expression pattern of *ABCC6P2* in 39 human tissues was highly similar to that of *ABCC6* and *ABCC6P1* suggesting similar regulatory mechanisms for *ABCC6* and its pseudogenes.

## Findings

### Background

**Figure 1:**
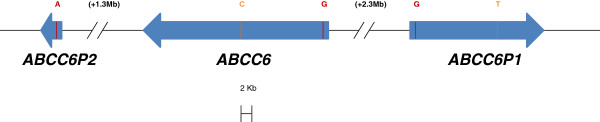
**Genomic organization of *ABCC6*, *ABCC6P1 *and *ABCC6P2 *. ***ABCC6 * is located on chromosome 16p13 between its two pseudogenes, *ABCC6P1* and *ABCC6P2*, at a distance of 2.3 Mb and 1.3 Mb, respectively. The boxes indicate the size of the genes and the arrows indicate the direction for transcription. Nucleotide difference in exon 2 between *ABCC6* (G), *ABCC6P1* (G) and *ABCC6P2* (A) is indicated in red colour while nucleotide difference in intron 7 between ABCC6 (C) and ABCC6P1 (T) is indicated in orange colour.

The ATP-binding cassette transporter ABCC6 belongs to a large family of membrane proteins (ABC transporters) that are a highly conserved and present in all organisms from bacteria to man [1,2]. The *ABCC6* gene (Entrez Gene ID 368) is located on the short arm of chromosome 16 along with two shorter, almost identical (> 99% sequence identity), pseudogenes; *ABCC6P1* (Entrez Gene ID 653190) and *ABCC6P2* (Entrez Gene ID 730013) (Figure 1) [3]. Pseudogenes are generally defined as non-functional genes, meaning that they usually do not produce a transcript or a functional protein [4,5]. Transcription of *ABCC6* pseudogenes have been described [3,6,7], and recently we found strong evidence for a regulatory interdependency between *ABCC6* and its pseudogene *ABCC6P1*[7].

Mutations and deletions in *ABCC6* are known to cause the rare (prevalence between 1:25,000 and 1:100,000), autosomal recessive disease pseudoxanthoma elasticum (PXE, OMIM 264800), a metabolic disorder characterized by ectopic mineralization of soft connective tissues [8,9]. *ABCC6* is located on chromosome 16, a known hotspot of chromosomal instability, showing several genomic duplications and deletions (generally called copy number variations) [10,11]. We therefore hypothesized that *ABCC6* pseudogenes would be liable to chromosomal rearrangements and thereby subject to copy number variations. Having less or more copies of *ABCC6* pseudogenes is likely to influence the expression level of these pseudogenes, and thus, may have an impact on the parent gene *ABCC6*, including the genetic message, the protein level and the function of the protein.

Estimation of copy number variations and transcription of pseudogenes is generally difficult because of the high sequence similarity between pseudogenes and their parent genes. However, pyrosequencing has recently shown to be a helpful tool to differentiate between highly similar genes by only one nucleotide difference [12]. Therefore, principally by the use of pyrosequencing, the aim of this study was to determine copy number variations of *ABCC6ABCC6P1* and *ABCC6P2*. Moreover, we sought to study the mRNA transcription pattern of *ABCC6ABCC6P1* and *ABCC6P2* in 39 different human tissues.

### Results

#### Copy number variations in *ABCC6* and its pseudogenes in human samples

Copy number variation of *ABCC6* was very rare (2/142; 1.4%) (Table 1) in healthy individuals. No individuals had deletions of *ABCC6*. Deviation in copy number was more frequent for *ABCC6* pseudogenes. In Chinese, deletions or duplications of *ABCC6P1* were more frequent than in any other population (9/24; 37.5%). Furthermore, in the total population (n = 142), one or three copies of *ABCC6P1* was relatively common (3% and 8%, respectively). Only one person had one copy of *ABCC6P2* while none had three copies. In Africans, however, no copy number variation was found for *ABCC6* pseudogenes. All the members of two Centre d’Etude du Polymorphisme Humain (CEPH) pedigrees had two copies of *ABCC6*, *ABCC6P1* and *ABCC6P2* (data not shown). As copy number variation was analyzed in short specific regions of *ABCC6* and *ABCC6* pseudogenes, small deletions/insertions in other regions of these genes cannot be excluded.

**Table 1:** Copy number variation in different populations

**Genes**	**Copies**	**Caucasians (N = 50)**	**Mexicans (N = 24)**	**Middle-East (N = 20)**	**Africans (N = 24)**	**Chinese (N = 24)**	**Total (N = 142)**
		**N (%)**	**N (%)**	**N (%)**	**N (%)**	**N (%)**	**N (%)**
*ABCC6*	1	0 (0)	0 (0)	0 (0)	0 (0)	0 (0)	0 (0)
	2	50 (100)	23 (96)	20 (100)	24 (100)	23 (96)	140 (99)
	3	0 (0)	1 (4)	0 (0)	0 (0)	1 (4)	2 (1)
*ABCC6P1*	1	0 (0)	0 (0)	1 (5)	0 (0)	4 (17)	5 (3)
	2	47 (94)	23 (96)	17 (85)	24 (100)	15 (62)	126 (89)
	3	3 (6)	1 (4)	2 (10)	0 (0)	5 (21)	11 (8)
*ABCC6P2*	1	0 (0)	0 (0)	0 (0)	0 (0)	1 (4)	1 (1)
	2	50 (100)	24 (100)	20 (100)	24 (100)	23 (96)	141 (99)
	3	0 (0)	0 (0)	0 (0)	0 (0)	0 (0)	0 (0)

#### Gene expression of *ABCC6* and its pseudogenes in human samples

**Figure 2:**
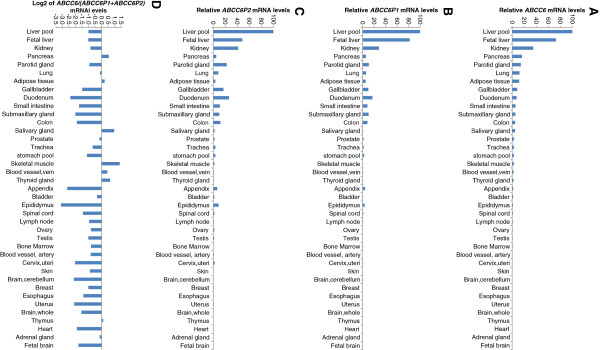
**mRNA expression of *ABCC6 *and its pseudogenes *ABCC6P1 *and *ABCC6P2 *in a variety of human tissues.** Relative normalized transcription of *ABCC6* (**A**), *ABCC6P1* (**B**) and *ABCC6P2* (**C**) in 39 human tissues. The tissue with the highest expression (liver pool) was used as reference tissue (100%). (**D**) Expression of *ABCC6* relative to *ABCC6P1* and *ABCC6P2*. Ratios are shown as a logarithmic scale (base 2).

*ABCC6* and *ABCC6* pseudogene expression profiling was performed in various human tissues (not corresponding to individuals analyzed for copy number variations). The transcription pattern of *ABCC6P2* was highly similar to *ABCC6* and *ABCC6P1*, with highest transcription in liver and kidney (Figure 2 A-C). Interestingly, the total mRNA level of *ABCC6* pseudogenes, *ABCC6P1* + *ABCC6P2*, was higher than *ABCC6* mRNA levels in most tissues, including liver and kidney (Figure 2 D). However, it should be noted that in tissues with low *ABCC6* mRNA levels, differences in expression between *ABCC6* and its pseudogenes may result from stochastic effects during the PCR.

### Discussion

In this study, we found copy number variation of *ABCC6* pseudogenes to be frequent, especially in populations of Chinese ancestry. However, as expected in healthy populations, no individuals had deletions of *ABCC6. ABCC6* and its pseudogenes, with their high sequence identity (>99%), represent a low copy repeat (LCR). LCRs are paralogue segments of usually >10 kb with >97% sequence identity and can act as substrates for nonallelic homologous recombination which may lead to deletion, duplication or inversion of the intervening sequence [13,14]. Phylogenetic trees on human and chimpanzee sequences show that *ABCC6* pseudogenes have occurred independently several times in these species, which further demonstrate the high mobility of these genes [15]. Our results confirm the hypothesis that *ABCC6* pseudogenes would be liable to genomic instability and thereby copy number variations.

Six percent of Caucasians are expected to have three copies of *ABCC6P1* (Table 1). However, none of the 35 members of two 3-generation-pedigrees (CEPH pedigrees) had deviation from the normal copy of two of *ABCC6* or *ABCC6* pseudogenes, indicating a Mendelian transmission of these copy numbers and that de novo deletion/duplication of pseudogenes do not arise frequently.

*ABCC6* and its pseudogenes share highly similar proximal promoter sequences (> 98.5% sequence identity) [7]. Furthermore, the hepatocyte nuclear factor 4α (HNF4α) binding site located at −166/-154 of the *ABCC6* promoter, which is crucial for tissue-specific expression pattern of *ABCC6*[16,17], is also present in the *ABCC6P1* and *ABCC6P2* promoters. Thus, the finding of similar expression pattern for *ABCC6* and both pseudogenes in human tissues strongly imply similar regulatory mechanisms for *ABCC6* and its pseudogenes. On the other hand, these results also suggest that both *ABCC6*- and *ABCC6* pseudogene transcripts have similar half-lives, which is surprising since they do not share the same mRNA 3’-ends. Pseudogenes were for a long time assumed to be “junk DNA”. However, recent studies have shown that many pseudogenes are functionally active and that they may influence their parent gene [7,18-23], and potential mechanisms of pseudogene function have been suggested. Copy number variation in pseudogenes has previously also been identified for the Neutrophil cytosolic factor 1 (*NCF1)* pseudogenes [24-26]. *NCF1* is a component of NADPH oxidase and having fewer or more copies of *NCF1* pseudogenes seem to influence the production of reactive oxygen intermediates [24,26]. It would therefore be interesting to investigate whether different copy numbers of *ABCC6* pseudogenes also influences the expression of the *ABCC6* gene. Unfortunately, the expression of *ABCC6ABCC6P1* and *ABCC6P2* in various human lymphoblastoid cell lines with one, two or three copies of *ABCC6P1*, which could be used for these studies, was too low to be detected by reverse transcription - quantitative real-time PCR (RT-qPCR) or pyrosequencing (data not shown).

### Methods

#### Human samples

Genomic DNA from the National Institute of General Medical Science (NIGMS) Human Variation Panels was purchased from the Coriell Cell Repositories (Camden, USA): The Caucasian Panel (n = 50); The Han People of Los Angeles Panel (n = 24); The Middle Eastern Panel, version 1 and 2, (n = 20); The Mexican-American Community of Los Angeles Panel (n = 24), The African-American Panel (n = 24) and two pedigrees (CEPH/Utah Pedigree 1331 and CEPH/Amish Pedigree 884). High quality RNA samples of the Human Total Master panel II and the Human Adult Normal Tissue Total RNA (39 tissues in total) were purchased from Clontech (Mountain View, USA) and BioCat Gmbh (Heidelberg, Germany) respectively.

#### Copy number variation analysis

For absolute copy number determination of *ABCC6*, the TaqMan® Copy Number Assay was used targeting *ABCC6* specifically in intron 11 (Hs03952142_cn; Applied Biosystems, Foster City, USA). Rnase P, which is known to be present in two copies in the human genome, was used as endogenous reference gene (TaqMan® Copy Number Reference Assay Rnase P, Applied Biosystems). The *ABCC6* assay (labeled with FAM), the RnaseP assay (labeled with VIC), sample DNA and 2xTaqMan Universal PCR Master Mix was combined in 20 μl reactions and ran in quadruplicate on a 7900HT Fast Real-Time PCR System using standard conditions (Applied Biosystem). The absolute copy number of *ABCC6* was thereby calculated using CopyCaller™Software v1.0 (Applied Biosystems).

For pyrosequencing, two sets of PCR primers were designed to amplify *ABCC6*, *ABCC6P1* and *ABCC6P2*. The first set of primers targeted intron 7 of *ABCC6* and *ABCC6P1* only, while the other set of primers targeted exon 2 of all three genes (Table 2 and Figure 1). The genes were amplified from ~100 ng of genomic DNA in 25 μL reactions using 1 x PyroMark PCR Master Mix, 1 x CoralLoad Concentrate (Qiagen, Venlo, The Netherlands) and 0.2 μM primers. Cycling conditions were an initial enzyme activation step at 95°C for 15 min and 45 cycles of 94°C for 30 s, 60°C for 30 s, and 72°C for 30 s, and a final extension cycle of 72°C for 10 min. Twenty micro liter of PCR products were added to 40 μL Binding Buffer (Qiagen), 2 μL streptavidin sepharose high-performance beads (GE Healthcare, Little Chalfont, United Kingdom) and 18 μL water and stirred for 5–10 min at 1400 rpm on a mixer. Single stranded biotinylated templates were isolated using PyroMark Vacuum Prep WorkStation (Qiagen) and dispensed onto PyroMarkQ24 plate containing 25 μL of 0.3 μM sequencing primer and Annealing Buffer (Qiagen). The plates were incubated for 2 min at 80°C and subsequently cooled at room temperature for at least 5 min. Sequencing was performed using a PyroMark Q24 instrument with PyroGold reagents (Qiagen). In the sequencing reaction, one and one nucleotide is added at the time, and peak heights are propotional to the amount of nucleotide molecules incorporated. Therefore, relative copy numbers were calculated using ratios of pyrogram peak heights: *ABCC6*/(*ABCC6* + *ABCC6P1*) = C5/C8 (C5 and C8 being the peak heights at dispensation 5 and 8, respectively for assay #1) and *ABCC6P2*/(*ABCC6* + *ABCC6P1* + *ABCC6P2*) = T8/T6 (T6 and T8 being the peak heights at dispensation 6 and 8, respectively for assay # 2, note; reverse sequencing) (Table 2).

**Table 2:** Primer sequences and nucleotide dispensation order used in pyrosequencing assays

**Assay #**	**Genes**	**Template**	**Primer**	**Sequence**	**Dispensation order**	**Amplicon size (bp)**
					**123456789**	
1	*ABCC6-ABCC6P1*	gDNA	Forward	5’-TGAGGGAGCCAGGCTAGA-3’		129
			Reverse	5’-Biotin-GAGGGGAAGGGAGAGATTAGC-3’		
			Sequencing	5’-GCCTGGCCCTGCCGC-3’	GTAGCTGCT	
2	*ABCC6-ABCC6P1-ABCC6P2*	gDNA	Forward	5’-Biotin-TCCCATCTACCTCCTCTTCATC-3’		76
			Reverse	5’-ATCTTGGCTTTGAAGAGTGG-3’		
			Sequencing	5’-TGGCTTTGAAGAGTGG-3’	CGACATCT	
3	*ABCC6-ABCC6P1-ABCC6P2*	cDNA	Forward	5’-Biotin-CGGGGCAGGGGGTCTGGAAC-3’		195
			Reverse	5’-ATCTTGGCTTTGAAGAGTGG-3’		
			Sequencing	5’-TGGCTTTGAAGAGTGG-3’	CGACATCT	

The absolute copy number for each allele (*ABCC6*, *ABCC6P1* and *ABCC6P2*) was finally deduced from the TaqMan® Copy Number Assay and the two pyrosequencing assays.

#### Gene expression analysis

cDNA was synthesized from total RNA from 39 tissues (1 μg) by reverse-transcription using Omniscript RT kit (Qiagen) in the presence of oligo-dT and random hexamer primers (Applied Biosystems) in 20 μL reactions.

Previously, the mRNA expression pattern of *ABCC6* and *ABCC6P1* in 20 different tissues was described by our group [7]. In this study, we determined the mRNA expression pattern of *ABCC6* and *ABCC6P1* in a total of 39 tissues by RT-qPCR on a 7900HT Fast Real-Time PCR System (Applied Biosystems). Five of twelve reference genes were estimated by geNorm [27] to give the most reliable normalization factors (see [28] for assay IDs): *GAPDH* = *PGK1* > *SDHA* > *CTBP1* > *GOLGA1* . The expression of *ABCC6* and *ABCC6P1* was normalized to these factors for each tissue.

Due to methodological issues, the mRNA expression pattern of *ABCC6P2* was not investigated in our previous study [7]. In this study, the relative mRNA expression pattern of *ABCC6P2* was analyzed by pyrosequencing. Primers for mRNA expression were designed to amplify *ABCC6ABCC6P1* and *ABCC6P2* expressed genes (Table 2). The relative transcription of *ABCC6P2* to *ABCC6* and *ABCC6P1* in the same 39 tissues was quantified using the same equation as above: *ABCC6P2*/(*ABCC6* + *ABCC6P1* + *ABCC6P2*) = T8/T6; Assay #3, Table 2), and by using the expressional data (RT-qPCR) of *ABCC6* and *ABCC6P1*. All PCR samples were run in parallels. No parallels varied more than 0.5 quantification cycles in qPCR experiments or 0.1 in pyrosequencing peak height ratios.

### Availability of supporting data

The data set supporting the results of this article are included within the article in Additional file 1 Table S1.

## Competing interests

The authors declare that they have no competing interests.

## Authors’ contributions

MKK, AP and JPB participated in the study design. MKK, CS and RMG carried out the experimental work. MKK and CS did the interpretation of data. MKK drafted the manuscript with assistance from CS and AP. All authors read and approved the final manuscript.

## Supplementary Material

Additional file 1: Table S1 Copy numbers of ABCC6, ABCC6P1 and ABCC6P2. Click here for file
